# Design and Implementation of an Interactive Website to Support Long-Term Maintenance of Weight Loss

**DOI:** 10.2196/jmir.931

**Published:** 2008-01-25

**Authors:** Victor J Stevens, Kristine L Funk, Phillip J Brantley, Thomas P Erlinger, Valerie H Myers, Catherine M Champagne, Alan Bauck, Carmen D Samuel-Hodge, Jack F Hollis

**Affiliations:** ^4^Duke University Medical CenterDurhamNCUSA; ^3^Johns Hopkins University School of MedicineBaltimoreMDUSA; ^2^Pennington Biomedical Research CenterBaton RougeLAUSA; ^1^Kaiser PermanenteCenter for Health ResearchPortlandORUSA

**Keywords:** Internet, website design, behavioral interventions, weight loss, weight maintenance

## Abstract

**Background:**

For most individuals, long-term maintenance of weight loss requires long-term, supportive intervention. Internet-based weight loss maintenance programs offer considerable potential for meeting this need. Careful design processes are required to maximize adherence and minimize attrition.

**Objective:**

This paper describes the development, implementation and use of a Web-based intervention program designed to help those who have recently lost weight sustain their weight loss over 1 year.

**Methods:**

The weight loss maintenance website was developed over a 1-year period by an interdisciplinary team of public health researchers, behavior change intervention experts, applications developers, and interface designers. Key interactive features of the final site include social support, self-monitoring, written guidelines for diet and physical activity, links to appropriate websites, supportive tools for behavior change, check-in accountability, tailored reinforcement messages, and problem solving and relapse prevention training. The weight loss maintenance program included a reminder system (automated email and telephone messages) that prompted participants to return to the website if they missed their check-in date. If there was no log-in response to the email and telephone automated prompts, a staff member called the participant. We tracked the proportion of participants with at least one log-in per month, and analyzed log-ins as a result of automated prompts.

**Results:**

The mean age of the 348 participants enrolled in an ongoing randomized trial and assigned to use the website was 56 years; 63% were female, and 38% were African American. While weight loss data will not be available until mid-2008, website use remained high during the first year with over 80% of the participants still using the website during month 12. During the first 52 weeks, participants averaged 35 weeks with at least one log-in. Email and telephone prompts appear to be very effective at helping participants sustain ongoing website use.

**Conclusions:**

Developing interactive websites is expensive, complex, and time consuming. We found that extensive paper prototyping well in advance of programming and a versatile product manager who could work with project staff at all levels of detail were essential to keeping the development process efficient.

**Trial Registration:**

clinicaltrials.gov NCT00054925

## Introduction

Obesity has become a major public health problem in the United States [1] with 65% of US adults now overweight or obese [2-4]. Obesity has been linked to increased overall mortality [5-7], decreased life expectancy [8,9], and greatly increased medical care costs [10-12]. The annual US medical expenditure attributable to obesity is estimated to be US $75 billion [13].

National recommendations for weight loss treatment call for intervention programs combining reduced energy intake, improved dietary choices, increased physical activity, and behavior therapy [14]. The most effective format for initial treatment is a series of weekly, professionally led group sessions [15-17]. Longer treatment programs result in greater weight loss, and many weight loss programs now continue initial treatment for 6 months. Immediate health benefits of weight loss include reduced blood pressure and improved blood glucose levels. Sustained reduction of even moderate amounts of weight (4 kg or more) has been shown to significantly reduce the risk of developing hypertension [18,19] and diabetes [20,21] over 3 years.

Even with successful weight loss during the first 6 months of treatment, there is a strong tendency toward weight regain following treatment termination. Although continuing weekly meetings as long as 40 weeks has been shown to be effective in preventing weight regain [17,22], a life-long series of weekly group meetings is not an attractive or practical option. To deal with this problem, there has been considerable interest in developing less intensive, but equally effective, long-term maintenance programs.

Recent reviews suggest that initial weight loss treatment may require different behavioral approaches than weight loss maintenance [15,17,23,24]. Specifically, building calorie counting skills and learning how to select less-energy-dense foods may be more critical for weight loss, whereas use of relapse prevention techniques, problem solving, and enhancing participant motivation may be more germane for weight maintenance. Due to their relatively low cost per person and flexibility of access, alternative communication technologies (eg, Internet) may provide attractive new channels for maintenance interventions [25-28]. For example, some studies comparing weight loss between an Internet-based intervention group and a therapist-led group found that weight loss was similar in the two groups [29]. Recent studies have shown that Internet-based weight loss interventions may be particularly cost-effective when trying to reach a large population [30].

The Weight Loss Maintenance Trial (WLM) (Trial Registration: clinicaltrials.gov NCT00054925) was designed to systematically study the efficacy of several different intervention strategies for helping participants maintain weight loss over a period of 2½ years. This paper describes the process by which the WLM research group designed and implemented one of these maintenance programs, featuring an Internet website and an associated prompting system using automated email and telephone messages, and the lessons learned during that process. Effectiveness data will be reported in a separate paper.

## Methods

### Design of the Weight Loss Maintenance Trial

The WLM is a four-center, randomized clinical trial testing the long-term efficacy of different strategies for maintaining weight loss. The design of the WLM is described elsewhere [31]. Briefly, participants in the WLM started a 6-month initial weight loss program focused on reducing caloric intake and increasing moderate intensity physical activity. Those who lost 4 kg or more were then randomly assigned to either a no-further treatment control condition or to one of two active weight loss maintenance interventions. The maintenance interventions were a personal contact condition, in which participants were contacted monthly by a health counselor, and an interactive technology (IT) condition, in which participants were encouraged to use an interactive website designed to help them maintain their weight loss. Weight loss results from WLM will not be available until mid-2008. The purpose of this paper is to describe the process by which the IT intervention program was developed, present utilization data for the first year, and provide a summary of what we learned from the development process.

#### Participants

We recruited adults with a body mass index (BMI) of 25-45 who were taking medication for either hypertension or hyperlipidemia. To be eligible, screening volunteers needed to have regular Internet and email access. Interested screening volunteers were sent an email message containing an individual identification number and the URL for a special screening website. Individuals needed to access that website and enter their identification number to be eligible for the study.

#### Weight Loss Interventions

This paper focuses on the development, implementation and use of one of the weight loss maintenance programs used in the WLM—the IT arm. Participants assigned to this arm used the website to record their weight, physical activity, and other weight loss activities. The website was designed to provide a number of important intervention elements, including social support using a bulletin board feature, record-keeping tools, tracking options, accountability, diet and exercise information, and tailored feedback.

### Website Design

Successful design strategies for the study’s interactive website can be summarized in three phases: (1) identifying the required skill sets for the design team, (2) specifying a stepwise process of designing the program, and (3) implementing the plan. Each phase is described in the following sections.

#### Design Team

**Figure 1:**
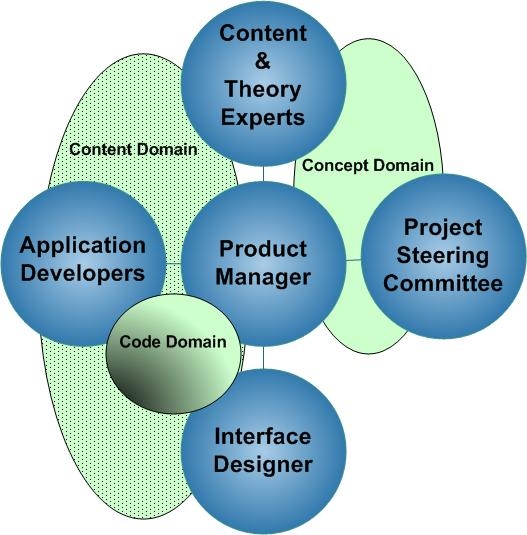
Overview of website design team; spheres and domains

Figure 1 displays an overview of the skills necessary for successfully designing an interactive behavior change website. Conceptual oversight, in this case the research project steering committee, determined the intervention’s overall goals and theoretical framework. The committee specified the objectives and scope of the website, set priorities and timelines, and kept current with related activities in other aspects of the research project. The role of the steering committee was to “think big” and provide scientific and conceptual guidance, but not to manage the project’s day-to-day implementation. Oversight decisions set the course for website design and, once determined, can be costly to reverse. The plans made by this group are “big picture” decisions, and documentation of these decisions is critical to the forward progress of website design. Allowing form and function details to distract this group can be a major pitfall.

WLM steering committee members included the principal investigators from a variety of specialty areas including psychology, cardiovascular health, epidemiology, nutrition, and clinical medicine. The theoretical framework chosen for this intervention combined self-directed behavior change theory [32,33], social support theory [34], motivational interviewing [35], and the transtheoretical stages of change model [36].

In addition to determining the intervention’s theoretical framework, the steering committee specified the overall objectives and scope of the website. They balanced the targeted outcome (in this case, using the website to maintain behaviors that promote maintenance of weight loss) with the available resources and the timeline for product development. Participants randomized to the maintenance phase of WLM had already achieved a minimum weight loss of 4 kg during a 20-session series of weekly group meetings. Thus, the website was designed for knowledgeable and successful participants with some experience in the application of behavior change techniques. Rather than building a website to prompt initial weight loss, our objective was to build a site to maintain and support existing behavior change habits while helping participants develop new self-management skills. Continued website activity by study participants has been identified as a concern in several website intervention studies [28,37-39], and keeping participants engaged over a long follow-up period (2½ years) was a key consideration in our design process. Finally, the aspect of social support was highlighted in the website’s overall objectives. Social support has been identified as a major supportive tool for continued behavior change [32,34]. Table 1 specifies the objectives of the WLM website.

**Table 1:** Objectives of the WLM interactive behavior change website

1. Reinforce existing behavioral self-management strategies
2. Facilitate and encourage new self-management skills
3. Improve self-efficacy for long-term weight management
4. Remain fresh and inviting to encourage regular, long-term contact
5. Promote social support among website users

Content and theory experts provide the scientific expertise necessary to translate the overall intervention goals into a website’s interactive modules. Content experts for the WLM website included master interventionists with graduate-level training in psychology, health counseling, nutrition, and physical activity. Building on the conceptual work of the research scientists on the steering committee, content and theory experts took the design process to a more detailed level. The input from content and theory experts ensured that the overall objectives were met in ways that were consistent with behavior change principles. The WLM content experts used their experience in conducting in-person weight loss interventions to identify key features of effective counseling sessions. Once identified, these key features were translated into interactive modules. Examples of key counseling features included offering choice, providing feedback, facilitating commitments to goals and plans, and minimizing the role of information while maximizing the importance of self-management. This group also determined strategies for implementing website modules that focused on key weight management behaviors (ie, encouraging participants to weigh themselves at least weekly). Weight entry was a “gatekeeper” to the home page. If a participant did not enter a weight upon log-in, the system directed the participant to the weight entry screen, leaving all other features disabled until a weight was entered. In a face-to-face counseling session for weight maintenance, there is a clear expectation that a weight will be taken and discussed during the visit, and this pattern was used during the 20-week initial weight loss program in WLM. Thus, requiring entry of weight at least weekly was not expected to be a barrier to website use. Making additional data entry requirements, however, was seen as a potential barrier for frequent website use.

The content experts translated the intervention’s objectives into specific plans and supplied most of the site content. The same background and skills needed to write an in-person curriculum are also needed when developing scripts for the interactive modules, but the automated systems have some constraints. For example, an in-person counseling session may be free flowing and touch on a variety of related and nonrelated issues before getting to the key counseling steps that move the participant toward a specific goal and action plan. Content for an interactive module, in contrast, must be at least conceived in a stepwise fashion. Because lifestyle change intervention counseling is an inherently iterative and tailored experience for both the counselor and the participant, developing and documenting content for use in an automated module is a challenge. See Table 2 for the steps used to develop interactive modules.

**Table 2:** Development of interactive modules

**Step**		**Participant Task**
1	Assess the situation	Identify the desired behavior change
2	Define the problem	Chose from a list of possible barriers
3	Determine a strategy	Decide on the best next step
4	Create a plan	Select one or more specific actions
5	Summarize and plan follow-up	Review a comprehensive plan and select a follow-up reminder date

The interface design specialists are the main contributors to the website’s look and feel. They also establish user functionality guidelines to be applied consistently throughout the website. The consultative and programming knowledge contributed by a user interface design expert is highly valuable and cannot be overlooked as an essential component of effective website design. A well-conceived module, based in sound behavior change theory and written with intensely rich content, will be of no value if the user’s experience is not considered during the design phase. Designing for a successful user experience considers details such as font style and size, balance of graphic and text, minimizing the “clicks” necessary to get to a desired place, and creating intuitive ways to navigate while simultaneously designing for wide variations in user hardware, software, and Internet service provider (ISP) limitations.

The application developers bring technical expertise unmatched by any other discipline. This role, simply stated, cannot be done by anyone but a skilled website developer. The developer group writes the website functionality code under the direction of the content experts. A labyrinth of behind-the-scenes systems support what appear to be simple modules. Multiple layers of system checks, error reports, data logging, and security measures exist within the programming of an interactive module, much of which is never seen by the larger design team or by website users.

The product manager serves as the communications hub for the design team and coordinates the entire design effort. As an indispensable member of the design team, the product manager must possess a variety of diverse skills and interests. Our experience developing the WLM website provided invaluable insights into the skills required for successful product management. These skills include the ability to contribute to conceptual oversight conversations while also being technically proficient to manage and evaluate minute details. The product manager must be skilled enough in each design team group to translate ideas effectively between groups. Additionally, the product manager must have the authority to make and finalize decisions. In our case, it was very helpful that the product manager (Funk) was also a content expert.

As with all teams, the groups of the website design team are interdependent. Whereas certain groups may interact frequently, others may interact only rarely. The “concept” domain (see Figure 1) includes the content and theory experts and the research scientists on the steering committee. A second domain, the “content” domain, includes the content and theory experts, applications developers, and user interface experts. A third “code” domain includes programmers and interface design experts. The product manager is a member of all domains. Other than the product manager’s bridging work, the workings of the concept domain need not intersect with the roles and functions of the content and code domains. This is where the role of a product manager becomes essential. The product manager forms the bridge between the high-level overviews from the concept domain to the much more detailed, linear, and literal language used in the coding domain. Too much interaction between nonconnected domains can lead to confusion, rework, and possible team dissatisfaction. The product manager keeps the boundaries clear between domains and facilitates communication between the groups.

#### Design Process

**Figure 2:**
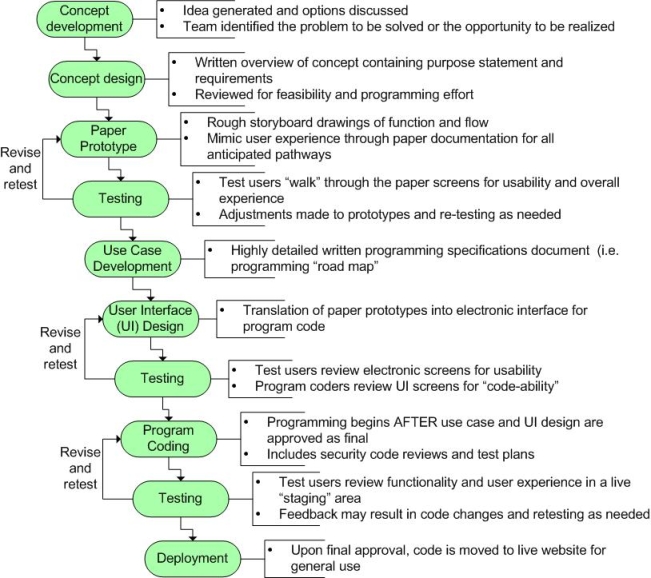
Steps in the website development process

The second phase of successful website design involves a clearly communicated stepwise process that outlines the development pathway of each interactive module. The design process described here assumes that the final website product is a unified collection of individually developed modules. The product manager maintains an ongoing record of each module’s progress (outlined in Figure 2). There are several key factors involved in successfully moving through the website development process. The concept design step requires a written purpose that provides a “compass” for future design considerations.

The development of a paper prototype is an equally important design step. Despite the tediousness of drawing out the “what if” scenario for every possible pathway, the paper prototype process is essential for efficient work. We learned that easily modified paper prototypes highlight unresolved problems before expensive programming time has been invested. Finally, delaying program coding until final approval of the use case (the detailed programming specifications) is imperative. Our experience developing this website taught us that programmers code exactly according to specifications. The more specific the use case, the more likely the product will be what the originators envisioned. We instituted a “sign off” step whereby the product manager signed off on the use case document prior to any programming. We believe this step helped to minimize rework by holding the originators accountable to their specifications.

#### Design Implementation

**Figure 3:**
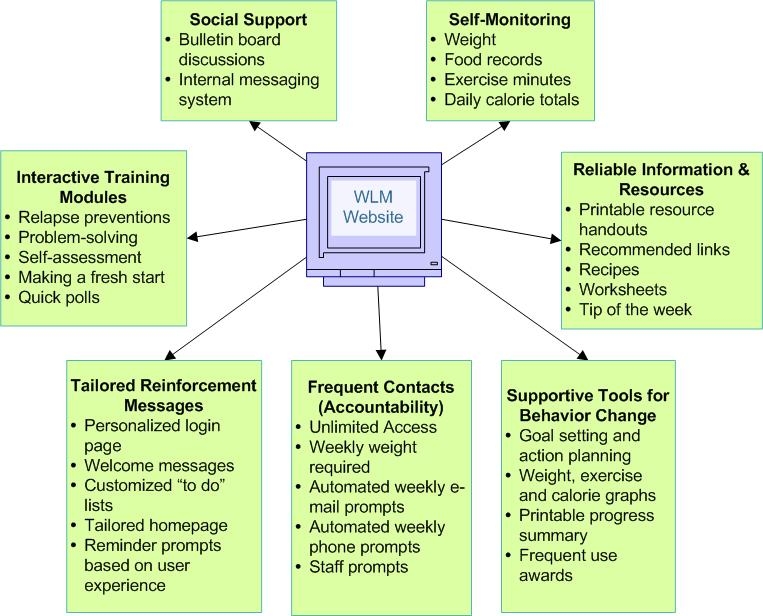
Overview of the WLM key interactive features

The WLM website’s key interactive features include social support, self-monitoring, written guidelines for diet and physical activity, links to appropriate websites, supportive tools for behavior change, check-in accountability, tailored reinforcement messages, and problem solving and relapse prevention training. An overview of the key interactive features is shown in Figure 3. Our implementation plan included 3 months of beta testing. We received feedback from 44 pilot participants as well as project staff. Feedback included comments posted on the beta testing discussion forum using the website bulletin board, emailed comments to the website moderator, and comments solicited by phone. Pilot participants were asked to log in to the website at least weekly and use all the website features. They were not required to meet the study eligibility criteria; however, many used the site to help with their own personal weight control. Our main feedback objective was to understand the user experience and what would enhance utilization of the website features.

**Figure 4:**
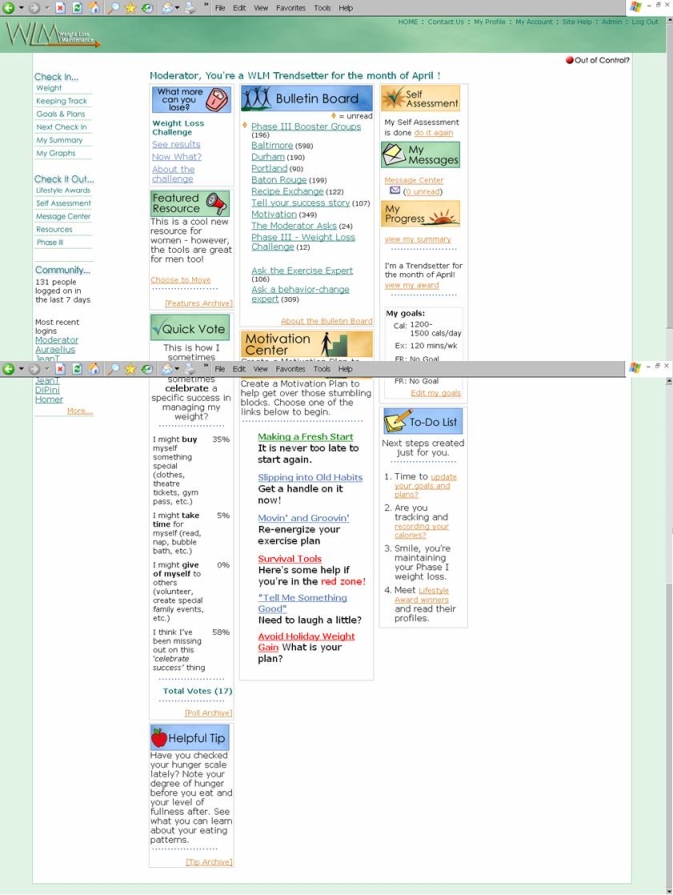
Sample screenshot of the website home page

**Figure 5:**
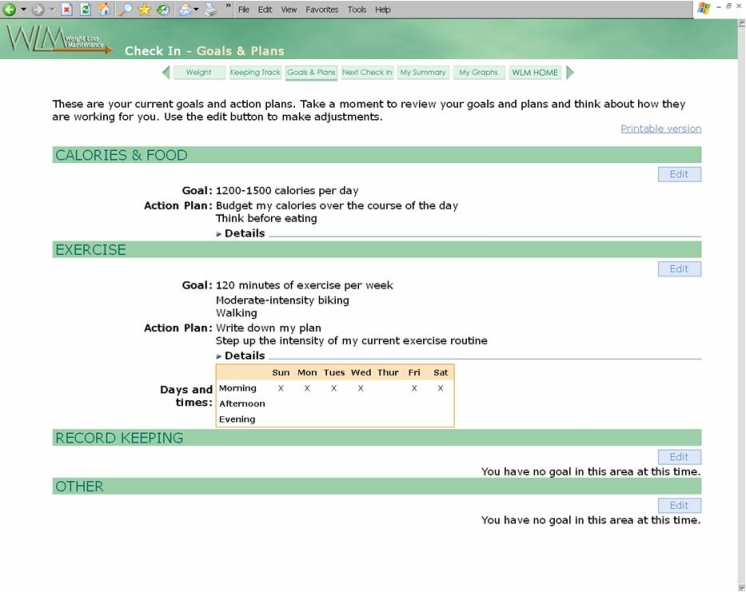
Sample screenshot of a participant’s goal setting page

A typical log-in experience included a tailored welcome message and the option to enter weight and diet information before proceeding to the home page. At the home page, participants could choose to engage in any number of the interactive features listed in Figure 3 or to log out. Given the website’s interactive nature and the intervention goal of weekly use for a 2½-year follow-up period, we learned from the beta testers that an individualized orientation to the website was much more likely to ensure user confidence and repeat log-ins than simply providing a website address and written instructions for use. Therefore, we instituted a participant orientation visit as part of our intervention protocol. Each participant was trained to use the website during an individual visit with a WLM staff interventionist. This training included an account setup during which the participant chose a display name, a first time check-in that demonstrated the usual weekly check-in expectations, and time to practise navigating the different features of the website, including an opportunity to post a message on the bulletin board. While the WLM participants had to pass a simple screening test for Internet access (receiving an email and visiting a special screening website), interventionists were trained to watch for specific technical barriers and to counsel accordingly during the orientation. Sample screenshots of the website home page and participant’s goal setting page are shown in Figure 4 and Figure 5, respectively. For a full overview of the WLM website screens, see the Multimedia Appendix.

### Prompting Use

**Figure 6:**
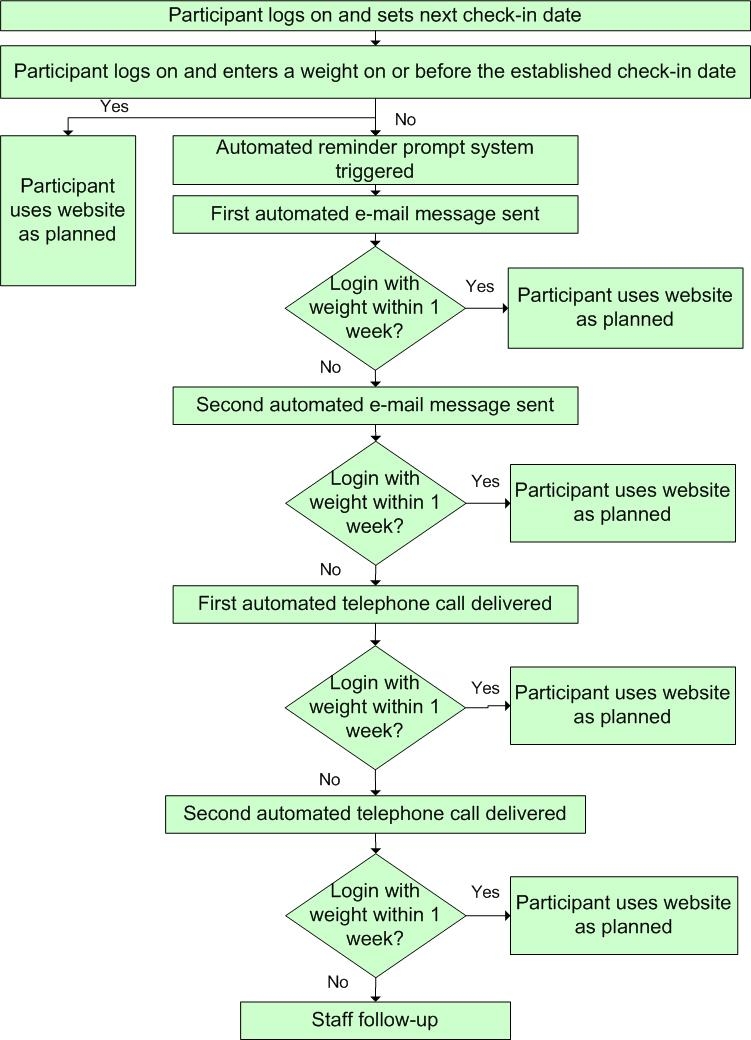
Overview of automated prompt system

Our weight loss maintenance program included an automated reminder system that prompted participants to return to the website if they missed their check-in date. An overview of the reminder system is shown in Figure 6. Specifically, if a participant had not logged on to the website on or before their next weekly check-in date, an automated email reminder was sent. Those participants who did not log on to the website within 1 week were sent a second email message prompt. Both email prompts were personalized, written in the spirit of motivational enhancement counseling (offering choice rather than instruction or advice), and contained a direct link to the website. If participants did not log on to the website within 1 week of the second email prompt, we employed automated telephone technology. This phone message was personalized and encouraged the participant to return to the website. A second automated telephone message was sent if the participant did not log in within 1 week of the first automated call. The system made every attempt to deliver the automated phone message, including multiple tries if the line was busy or hang-ups occurred. If there was no log-in response to the email and telephone automated prompts, a staff member called the participant. This individual effort continued until either the participant logged on to the website or the study ended.

## Results

Table 3 shows the characteristics of the 348 WLM participants assigned to the IT arm of the trial; 63% were female and 38% were African American. The mean age was 56 years, and the mean BMI at the start of the initial weight loss intervention was 34. IT participants lost a mean of 9 kg during the initial weight loss treatment.

**Table 3:** WLM IT participant characteristics (N = 348)

Mean age (years)	56
% female	63
% African American	38
Mean BMI at start of phase I	34
Mean weight loss in phase I (kg)	9

**Figure 7:**
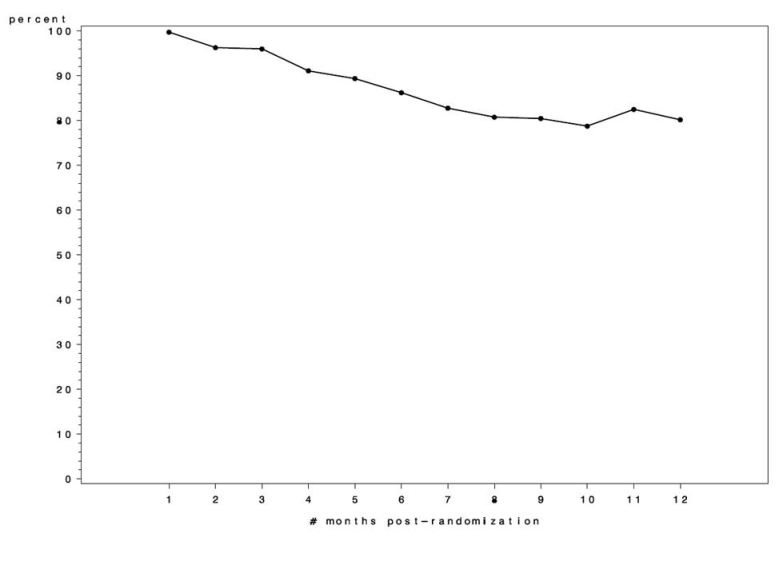
Percent of participants with at least one log-in per month

Weight loss data from the trial will not be available until mid-2008, but preliminary data on website use during the first year following randomization are presented here. During the first year, active website use (defined as at least one log-in per month) remained high, with over 80% of the participants still using the website in month 12 (Figure 7). In other words, approximately 20% of participants were no longer active users of the website after 12 months of the intervention. Furthermore, during the first 52 weeks, participants averaged 35 weeks with at least one log-in.

Table 4 shows the cumulative percentage of log-ins occurring as a result of the automated reminder prompt system: 86.4% of the first weekly email prompts resulted in the participant logging in to the website within 1 week, and 56.7% of the second email prompts resulted in a log-in within 1 week. Overall, the escalating series of email and automated telephone calls effectively prompted participants to return to the website in 97.3% of cases.

**Table 4:** Log-ins as a result of the automated prompts (first 12 months)

**Prompt**	**No.**	**Logged In After Prompt (%)**	**Cumulative Percent**
1st auto email	9372	86.4	86.4
2nd auto email	1278	56.7	94.1
1st auto phone call	554	36.8	96.3
2nd auto phone call	350	26.9	97.3
Staff phone call	256	64.8	99.0
Never responded	90	0	100.0

## Discussion

Behavior change websites not only offer lower cost per participant than face-to-face contacts with counselors, but also hold promise as an unexplored new mechanism for supporting long-term behavior change. Our experience developing the WLM behavior change website shows that high rates of use can be maintained for at least 1 year. Participation in this study was much greater than in some other long-term behavioral interventions [40-42], but similar to that seen in the Stop-Regain trial [43].

We have identified several major lessons from our experience developing and implementing the WLM interactive website. First, it is essential to specify the theoretical foundation of the intervention program and the website objectives early in the design process. Website design does not iterate the same way as development of in-person, counseling-based interventions. Making clear decisions about intervention objectives and abiding by them during the design process helps eliminate costly rework.

Our second key learning was that detailed paper prototypes and specification documents should always precede programming. While the design team may want to jump directly to screenshots before the logic has been thoroughly outlined, the results tend to be better if they wait for the paper prototyping to be completed. In the WLM, we called paper prototype meetings “wall meetings” because hours were spent taping freehand paper “screens” to the wall and determining the outcome of every link. These wall meetings resulted in many modifications and intervention improvements that would have been difficult during later stages in the development process. The final set of freehand paper screens was also very useful when writing the detailed specifications (use case) for the programmer.

A final key learning was to not underestimate the essential role of a product manager. To have all groups doing what they do best requires a central team member to intersect with each group. We believe that the product manager must be able to manage in all three domains (concept, content, and code) to ensure an effective website design process. The product manager helps to keep the development team working toward a common goal while serving as a translator for those working at different levels of detail.

Additionally, we gained insight into participant use of an interactive website and what is required to keep participants engaged. Based on preliminary “hit” counts (data not shown), interactive features like the weight entry form and the bulletin board discussion appeared to be most popular. We were concerned that a website intervention would have problems maintaining the interest of participants for long-term follow-up. We found, however, that a high rate of participation can be achieved for at least 12 months with an interactive website. The automated email and telephone reminders were quite effective in prompting regular use of the website for at least 1 year. In an 8-week, stage-based physical activity website intervention, Leslie et al [39] determined that emails prompted return visits to a website (77% returned after email prompts), but that the same emails were not helpful for encouraging new users to visit for the first time. In contrast to the Leslie et al study, participants in WLM were initially oriented to the website through a 1-hour individual visit with an interventionist. Personalized website orientation may be a critical factor in the effectiveness of subsequent email prompts that encourage returning to the website. Another study [44] reported that well-constructed email messages can have a beneficial effect on diet and exercise health behaviors. The authors suggest, however, that the email messages may need further tailoring and grounding in health behavior change theory to strengthen their potential. The WLM email prompts were customized to the individual user, easy to read, provided choice rather than advice, and included a link for easy access to the WLM website. These factors were potentially positive contributors to the effectiveness of our email prompts. We were initially concerned about the number and frequency of email reminders sent to study participants. We only sent email prompts when the participant met the specified criteria of not logging on to the website within 1 week of the last log-in. However, during the first 6 months of the intervention, 83% of participants received a weekly email prompt to log on to the website, providing us with two important lessons: (1) participants are not bothered by reminders to return to the website, and (2) in general, participants do not set up outside reminders to log in, but simply wait to be prompted.

Finally, it is important to note the current limitations of Web-based programs. Developers of behavior change websites must be prepared to continually update the product and limit the use of available technologies in consideration of bandwidth limitations. Danaher et al [45] urge developers to consider the bandwidth necessary to operate rich-media websites as a possible barrier to participant use. User frustration resulting from long page downloads presents a near terminal problem for researchers looking to test behavioral website use. Therefore, we limited the bandwidth requirements of the WLM website to accommodate those with limited bandwidth. For this reason, the WLM website is devoid of photos, moving text, video clips, and music. Also acknowledged by Danaher et al [45], the scalability of a behavior change website must be considered at the time of development. The capacity of a website to “grow” beyond its current capacity is an essential consideration.

Given our study timeline, we developed and implemented this website in 12 months. Looking back on our experience, additional development time would have been beneficial in three key processes: (1) at least 6 months of general beta testing (we had 3 months), (2) an even richer understanding of the user experience from the pilot participants (ie, periodic individual interviews to understand how/if the user experience evolved over time), and (3) additional opportunities to test multiple prompting strategies for encouraging participants to continue using the site. Even without such additional development time, use of this website remained high throughout the first year, with over 80% of the participants continuing to be active users during the 12th month.

## Multimedia Appendix

Sample screenshots of the WLM interactive website

## Abbreviations

BMI: body mass index
IT: interactive technology condition
WLM: Weight Loss Maintenance Trial
